# HER3 targeting potentiates growth suppressive effects of the PI3K inhibitor BYL719 in pre-clinical models of head and neck squamous cell carcinoma

**DOI:** 10.1038/s41598-019-45589-y

**Published:** 2019-06-24

**Authors:** Kara S. Meister, Neal R. Godse, Nayel I. Khan, Matthew L. Hedberg, Carolyn Kemp, Sucheta Kulkarni, Diego Alvarado, Theresa LaVallee, Seungwon Kim, Jennifer R. Grandis, Umamaheswar Duvvuri

**Affiliations:** 1 Department of Otolaryngology—Head & Neck Surgery, University of Pittsburgh Medical Center, Eye and Ear Institute, Suite 500, 200 Lothrop St., Pittsburgh, PA 15213 USA; 2 Kolltan Pharmaceuticals, New Haven, CT USA; 3 Department of Otolaryngology—Head & Neck Surgery, University of California—San Francisco, San Francisco, CA USA

**Keywords:** Head and neck cancer, Cancer genetics

## Abstract

BYL719 is a PI3K inhibitor that has demonstrated efficacy in the treatment of head and neck squamous cell carcinoma. BYL719 exerts its therapeutic effect by suppressing AKT and other proliferative signaling mechanisms. Despite PI3K inhibition and AKT suppression, residual activity of protein S6, a core marker of proliferative activation, has been observed. HER3, either via dimerization or activation by its ligand neurgeulin (NRG), is known to activate PI3K. Thus, we hypothesized that co-targeting HER3 and PI3K would lead to greater suppression of the PI3K-AKT signaling pathway and greater tumor suppression than with BYL719 alone. We investigated biochemical expression and activation of the HER3-PI3K-AKT-S6 pathway in HNSCC cell lines and patient-derived xenografts (PDXs). Antitumor effects of HER3 and PI3K inhibitors alone and in combination were evaluated in cell culture and murine models. Treatment of HNSCC cell lines with BYL719 significantly reduced AKT activation and suppressed tumor growth. However, S6 was persistently activated despite suppression of AKT. Combination treatment with KTN3379, a monoclonal antibody targeted against HER3, and BYL719 led to enhanced suppression of *in vitro* and *in vivo* cancer growth and durable suppression of AKT and S6. Therefore, inhibition of HER3 with KTN3379 enhanced the effects of PI3K inhibition in pre-clinical HNSCC models. These data support co-targeting HER3 and PI3K for the treatment of HSNCC.

## Introduction

Cancer is marked by numerous genetic mutations, of which a subset of “driver” mutations contribute to tumor progression and cancer phenotype(s). The driver mutations and their consequences in HNSCC, a frequently lethal malignancy, are incompletely understood^1,2^. Increased understanding of these mutations and their contributions to the landscape of HNSCC pathology will facilitate development of anti-cancer therapeutics and identify biomarkers to predict which patients are most likely to have an efficacious response to driver-targeted treatments.

In the HNSCC oncogenome, the mitogenic entity PI3K is altered in up to 80% of HNSCC^1,3^. Recent mutational profiling of HNSCC tumors by our group and others indicates that *PIK3CA*, a component of PI3K, is the most commonly known oncogenic driver in HNSCC; specifically, gain-of-function mutations in *PIK3CA (*6%–13%), *PIK3CA* gene overexpression (52%), and *PIK3CA* amplification (20%)^3,4^. BYL719 is a small molecule PI3Kα-selective inhibitor that has shown modest efficacy in treating advanced solid tumors, including HNSCC, in early-stage clinical trials^5,6^.

HER3 (ErbB3) has recently been touted as a major link between receptor tyrosine kinases and PI3K pathway activation due to having 6 binding sites for PI3K binding compared to the more common single binding site. High HER3 expression has been correlated with poor overall survival in several subsets of patients with HNSCC and other cancer types^7–10^. Activation of HER3 proceeds via dimerization with other HER-family receptors and/or by binding the endogenous ligand, neuregulin (NRG). This leads to subsequent activation of downstream signaling pathways, including PI3K/AKT/mTOR^11–13^. KTN3379 is a human anti-HER3 mAb that has unique features that contribute to its potency including a novel binding epitope^14^ and a three amino acid substitution (YTE) in the Fc portion of the mAb to improve PK parameters^15^. KTN3379 is currently undergoing clinical investigation in HNSCC (NCT02473731)^16–19^.

It has been previously observed that, while BYL719 suppressed activation of AKT and led to tumor suppression, other downstream signaling targets like S6 demonstrated persistent activation^12^. Furthermore, PI3K pathway inhibition has been shown to result in upregulation of HER3 expression, a known driver of PI3K and AKT activity^16,17,20^. Thus, we hypothesized that co-targeting PI3K with BYL719 and HER3 with KTN3379 would provide more effective suppression of the PI3K-associated signaling and have synergistic anti-cancer activity^21,22^.

## Materials and Methods

### HNSCC cell culture

All human cell culture experiments and described methodology were performed under the guidelines and protocols established by the University of Pittsburgh Institutional Research Board (IRB). All cell lines underwent genotype verification by commercial SNP analysis within 6 months of use. The HNSCC cell line FaDu was obtained from American Type Culture Collection. PE/CA-PJ34 (clone 12) cells were obtained from Sigma-Adrich. Cal33 was a kind gift from Dr. Gerard Milano (University of Nice, Nice, France). Cal33 and FaDu cell lines were cultured in Dulbecco’s Modified Eagle’s Medium (DMEM, Corning/Mediatech, Inc., Herndon, VA). PE/CA-PJ34 (clone 12) cells were cultured in Iscove’s Modified Dulbecco’s Medium with L-glutamine and 25 mM HEPES (IMDM, Corning/Mediatech, Inc., Herndon, VA). All media contained 10% heat-inactivated fetal bovine serum (FBS), and 1% Pen/Strep (Life Technologies, Grand Island, NY). All lines were maintained at 37 °C with 5% CO_2_. Cell cultures were tested every 12 weeks for mycoplasma contamination.

### Reagents and pharmaceutical compounds

KTN3379, a human IgG1 mAb with YTE substitutions, and the control antibody KTN0062C, an anti-KLH chimeric IgG1 mAb were provided by Kolltan Pharmaceuticals. BYL719 (S2814), which selectively inhibits alpha isoform, was purchased from Selleck. BYL719 was dissolved in DMSO for cell culture experiments. Recombinant human neuregulin/heregulin-1 (NRG1-β1/HRG-β1) was purchased from R&D Systems (396-HB/CF), and reconstituted in sterile PBS.

### Western blotting

Cells were cultured in the indicated experimental conditions. Whole cell lysates were prepared with lysis buffer combined with protease and phosphatase inhibitor; protein concentration was estimated using Bradford’s method. Equal amounts of protein was denatured and separated on 6–8% SDS-PAGE gels with subsequent transfer to nitrocellulose membranes. Membranes were probed with the indicated primary antibodies (listed below) and then with secondary antibodies for use with the LiCor imaging system. All membranes were developed on the LiCor Odyssey imaging system. Densitometry was performed with software provided with LiCor Odyssey imaging system; signal strength was normalized to appropriate loading control (Beta-Tubulin or Beta-Actin). Any changes to images (adjusting contrast and brightness) were applied uniformly to the entire image to maintain image integrity. Images were cropped for conciseness in figures; full length blot images are available in supplementary materials.

The following antibodies were used: pHER3, HER3, pAKT, AKT, pS6, S6, Beta-Tubulin, Beta-Actin. Pertinent phosphorylation sites included P-HER3 (Y1289), P-HER2 (Y1248), P-EGFR (Y1173), and P-AKT (S473). All antibodies were obtained from Cell Signaling Technology. Complete Mini protease inhibitor cocktail and PhosStop phosphatase inhibitor were from Roche. Bradford’s protein estimation reagent and molecular weight markers were from BioRad.

### Cell proliferation assays

Cell proliferation was assessed with the CellTiter Glo reagent (Promega). Briefly, cells were plated in 96 well plates in 4% FBS-containing medium and treated with indicated compounds for 72 hours. IC_50_ values for growth inhibition were determined by comparing with control cells. Comparative data was analyzed with the signed rank test and tested the specific hypothesis that the log ratio = zero. All data was obtained as the average of three experiments, and was performed in triplicate. Combination Index values were determined using CalcuSyn based on the Chou-Talalay method^23^.

### Colony formation assays

Cell lines were treated with increasing doses of KTN3379, BYL719, and/or NRG1-β1 as indicated. Colony formation assays were stopped when any well reached ~80% confluence, ranging from 7–15 days. Colonies were fixed with 10% neutral buffered formalin solution and stained with 0.01% (w/v) crystal violet in dH_2_O. Quantification was performed via the ColonyCounter plug-in for ImageJ.

### HNSCC xenograft models

Animal care was in strict compliance with institutional guidelines established by the University of Pittsburgh Institutional Animal Care and Use Committee (IACUC), the Guide for the Care and Use of Laboratory Animals (National Academy of Sciences, 1996), and the Association for Assessment and Accreditation of Laboratory Animal Care International. PE/CA-PJ34 (clone 12, 2 × 10^6^ cells/injection)) cells were injected into the left and right flank, respectively, of non-obese diabetic/severe combined immunodeficient γ (NOD/SCIDγ) mice. Treatment began when tumors became palpable. Tumor formation was determined by palpation and measured with calipers 3 times weekly. Tumor volume was calculated by the following formula: volume = V = ½ × depth × (length)^2^; length = longest axis of measurement, with depth defined as a right angle to length. When tumor volume reached ~75 mm^3^, mice were randomly assigned to four groups (n = 7/group, n = 28 total), and treated with one of the following: vehicle control, BYL719, KTN3379 or BYL719 + KTN3379 for 4 weeks, or until tumor growth required sacrifice. Control group-mice received intraperitoneal (i.p.) injection of 100 µl phosphate-buffered saline (PBS). BYL719 was administered at 20 mg/kg daily for 5 days per week by oral gavage. Powdered BYL719 was solubilized in 1% (w/v) carboxymethylcellulose (CMC) + 0.5% (w/v) Tween 80, max administration volume = 200 ul/dose. KTN3379 was administered at 5 mg/kg i.p. injection twice weekly. The combination treatment group utilized administration of BYL719 (oral gavage, 5x/week, 20 mg/kg) + KTN3379 (i.p. injection, 2x/week, 5 mg/kg).

### Organ slides

Organs (heart, lung, kidney, liver, and spleen) were harvested from mice treated with vehicle control, BY719, KTN3379, and combination BYL719 and KTN3379. Tissue sections were prepared and stained with hematoxylin and eosin for gross observational analysis.

### *In-vivo* patient-derived xenograft (PDX) studies

Human tumors were obtained under the auspices and protocols established by the University of Pittsburgh IRB and the University of Pittsburgh IACUC after obtaining informed consent from the subject or parent/legal guardian. Studies were performed as previously described^24^. For the *in vivo* experiment, non-obese diabetic/severe combined immunodeficiency (NOD SCID) gamma mice (4–6 weeks old; 20 g; The Jackson Laboratory, Bar Harbor, Maine) were implanted subcutaneously with HPV negative, *PIK3CA* wildtype tumors from HNSCC patients. Once the tumors were palpable, mice were randomized and treated with BYL719 or PBS as a vehicle. At the end of the treatment period, tumors were harvested and lysates were collected for determination of protein expression. The tumor tissues were processed for Western blot analyses to evaluate expression of pAKT, pHER3, AKT, and HER3. β-Tubulin was also evaluated and used as a loading control.

### Statistical analysis

Error bars represent standard error of the mean and asterisks indicate statistically significant differences with groups compared by Student *t* test. In cases of multiple *t* tests, statistical significance was determined using the Holm-Sidak method, with α = 0.05. Survival analysis was performed using the Kaplan Meier method with SPSS software, version 21.0 (SPSS, Chicago, IL, USA). Group-wise comparisons were made using the log-rank test.

## Results

### PIK3CA inhibition leads to HER3 upregulation and activation

**Figure 1:**
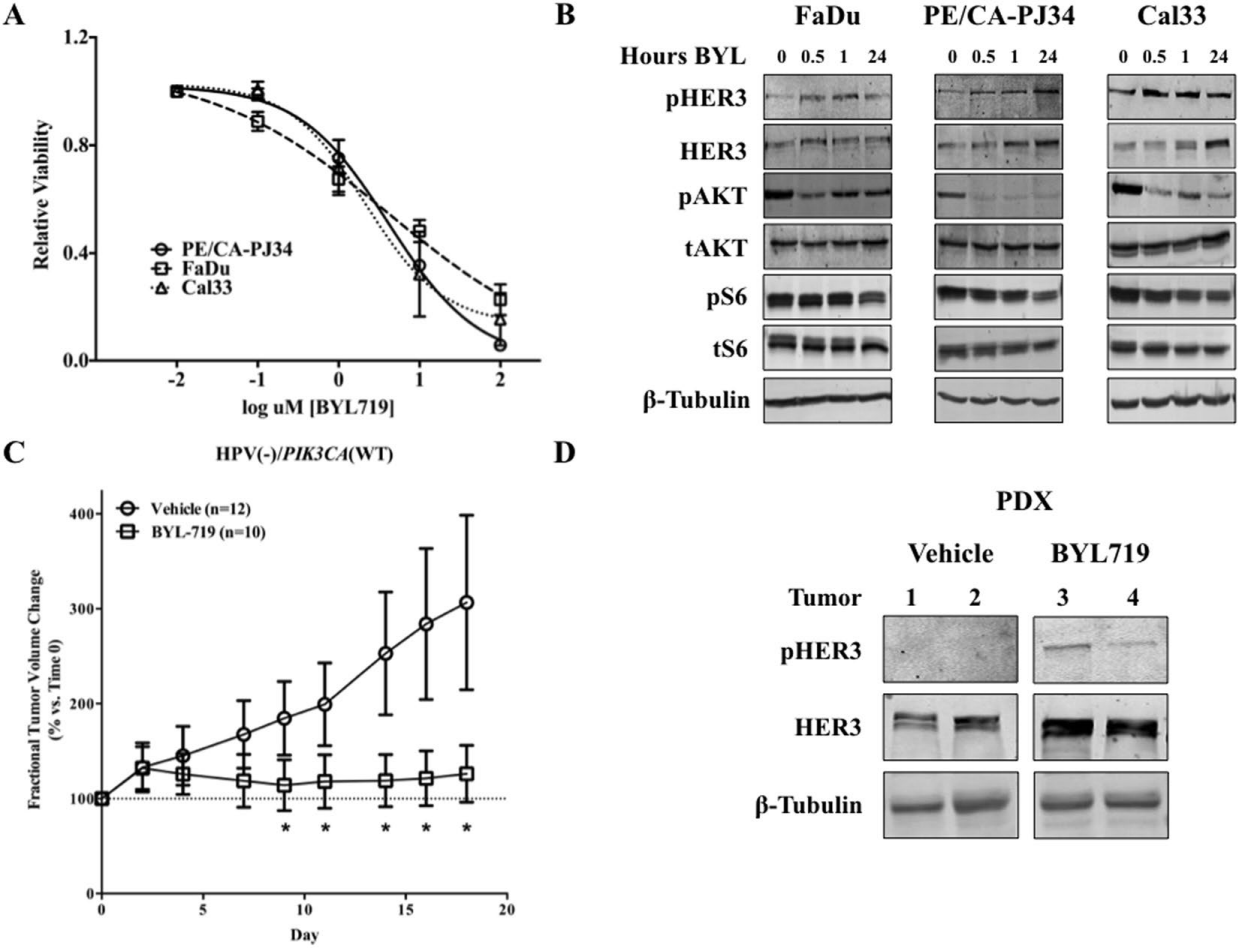
*PIK3CA* inhibition with BYL719 increases expression and activation of HER3. (**A**) *PIK3CA* inhibition with BYL719 inhibits HNSCC growth in multiple cell lines. (**B**) AKT is potently suppressed following *PIK3CA* inhibition with 1 µM BYL719 *in vitro*. Protein S6 remains persistently activated. (**C**) *PIK3CA*^*WT*^ patient derived xenografts (PDX) treated with BYL719 had significantly smaller volume than vehicle-treated tumors. (**D**) BYL719 treated PDXs (tumors 3 and 4) also demonstrated upregulation of HER3 relative to control tumors (tumors 1 and 2). Data presented as mean ± SEM unless as otherwise indicated; **p < 0.01. Blots cropped for conciseness; full length images available in Supplementary Data.

A panel of seven human HNSCC cell lines with varying PIK3CA and PTEN statuses were treated with BYL719 to assess the IC50 (Sup. Fig. 1). From this cohort, PE/CA-PJ34, FaDu, and Cal33 (*PIK3CA* wildtype, amplified, mutated, respectively) were selected for further study as representative cell lines. Treatment with BYL719 demonstrated consistent *in vitro* growth suppression in all three cell lines (Fig. 1A). Immunoblot analysis of cells treated with BYL719 for progressively longer periods of time demonstrated a consistent loss of AKT activity, but comparatively little loss of S6 activity (Fig. 1B). Of note, in PE/CA-PJ34 and Cal33 a time dependent increase in total HER3 expression was noted on immunoblot (Fig. 1B) following exposure to BYL719. This increase was specific to HER3 as a corresponding increase in other HER-family receptors, NRG, or MET was not observed under these conditions (Sup. Fig. 3).

Next, we used a patient derived xenograft (PDX) model to assess the *in vivo* effects of BYL719. A single HPV (-), *PIK3CA* wild type human tumor was propagated and split into two cohorts of mice - vehicle control (n = 12) or BYL719 (n = 10). PDX tumors treated with BYL719 were significantly smaller than control treated counterparts (Fig. 1C). Immunoblot analysis of two representative BYL719-treated PDX tumors also demonstrated upregulation of HER3 (Fig. 1D).

Taken together these data suggest that *PIK3CA p110α* inhibition with BYL719 suppresses AKT and has anti-tumor activity. However, in the presence of persistent activation of S6, the possibility of HER3-driven activation through other PI3K subunits remained.

### PIK3CA inhibition is partially reversible by NRG stimulation

To test the hypothesis that HER3-driven activation of the remaining PI3K subunits could activate proliferative pathways, we treated HNSCC cell lines with a combination of NRG, the endogenous ligand of HER3, and BYL719.

**Figure 2:**
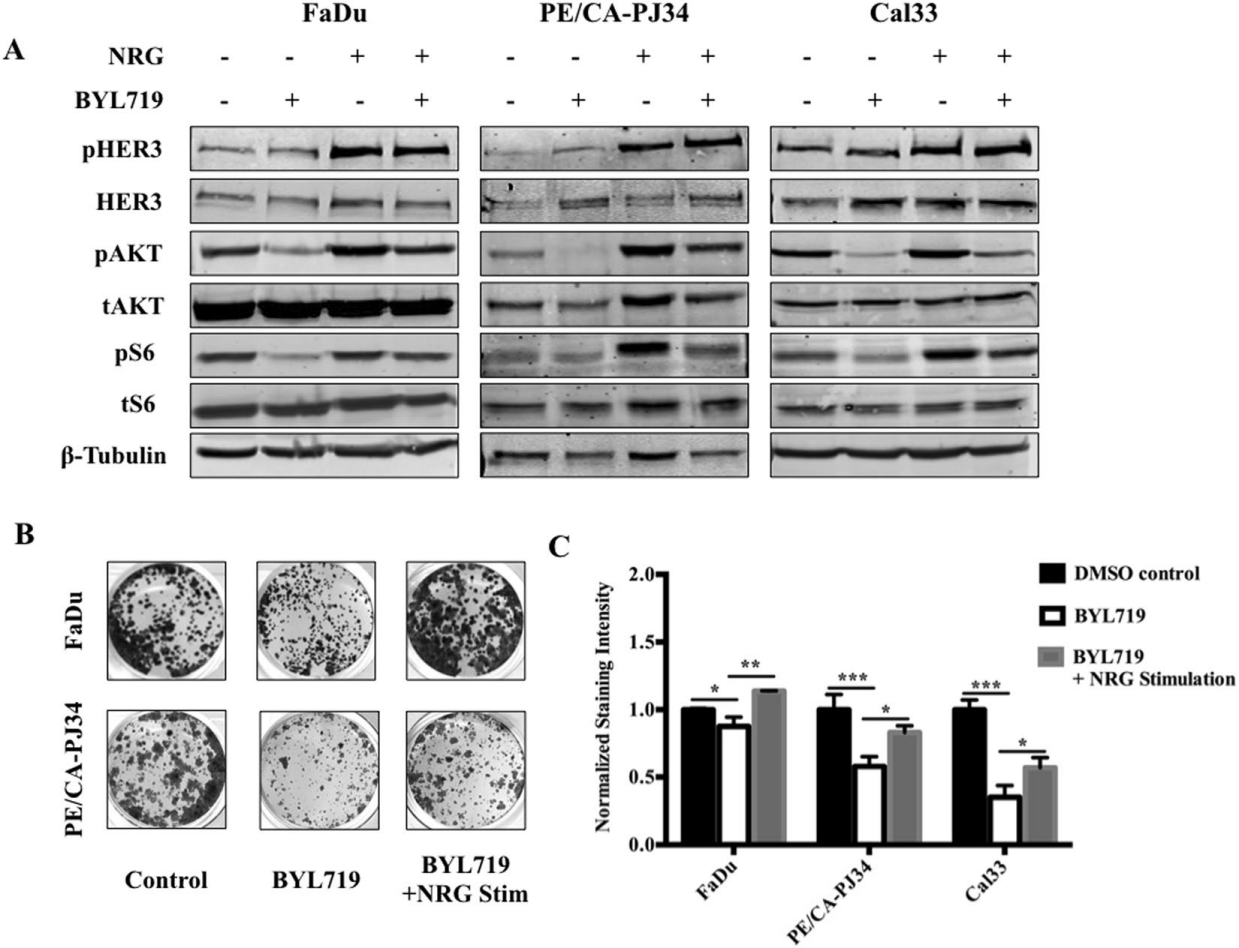
NRG stimulation can overcome BYL719-mediated growth inhibition. (**A**) Stimulation with 10 ng/ml NRG for 10 minutes partially overcomes downstream AKT signaling and enhances HER3 activation following treatment with 1 µM BYL719 (24 hour). (**B**) Treatment with BYL719 (1 µM) leads to growth inhibition that is partially reversible with NRG stimulation (10 ng/ml); representative colony formation assays presented. (**C**) Quantification of colony formation staining intensity in Cal33, FaDu, and PE/CA-PJ34 cell lines. Data presented as mean ± SEM unless as otherwise indicated; *p < 0.05, **p < 0.01, ***p < 0.001. Blots cropped for conciseness; full length images available in Supplementary Data.

Consistent with previous results, BYL719 treatment alone led to an increase in HER3, most prominently in PE/CA-PJ34 and Cal33 cells, while inhibiting downstream activation of AKT (Fig. 2A). However, the addition of NRG was able to overcome the changes induced by *PIK3CA p110α* inhibition with BYL719 - HER3, AKT, and S6 were all strongly activated, as shown by enhanced phosphorylated HER3 (pHER3) and phosphorylated AKT (pAKT) (Fig. 2A).

Next, we evaluated colony formation to assess whether our immunoblot findings corresponded to a functional consequence on tumor cell growth. Addition of BYL719 was able to strongly inhibit clonogenic potential; however this growth inhibition was overcome with the addition of NRG (Fig. 2B,C). These data demonstrate that activation of HER3 with NRG can lead to AKT and S6 activation even in the presence of PI3K p110α inhibition.

### A monoclonal antibody to HER3 suppresses NRG-dependent signaling and HNSCC proliferation

**Figure 3:**
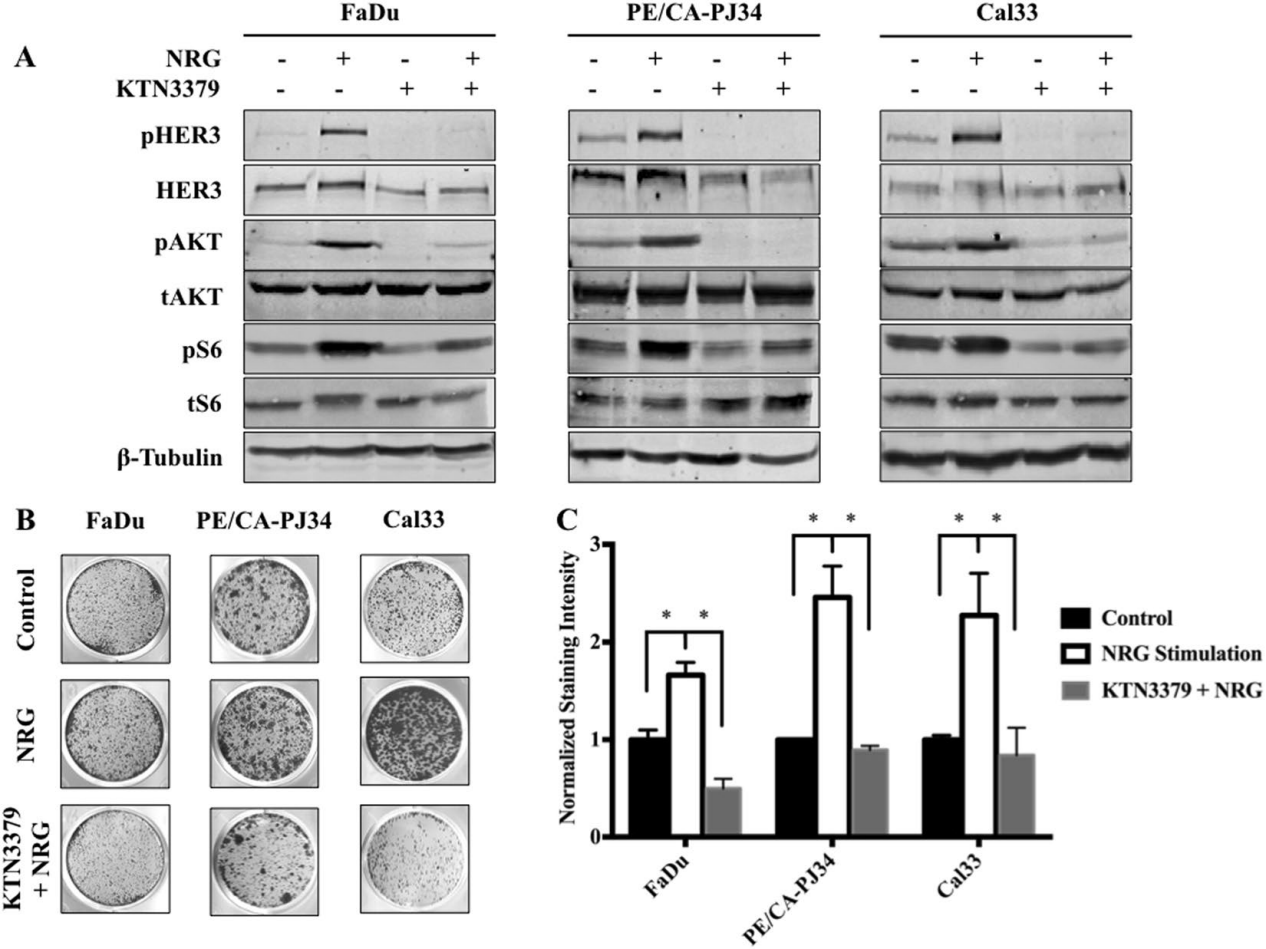
HER3 inhibition prevents NRG-mediated HER3-AKT activation. (**A**) NRG stimulation (10 ng/ml, 10 min)-induced activation of HER3-AKT is reversible with KTN3379 (50 nM, 1 hour) treatment in FaDu, PE/CA-PJ34, and Cal33 HNSCC cells. (**B**) Stimulation with NRG (10 ng/ml) promotes colony formation; this effect is abrogated with addition of KTN3379 (100 nM). Representative colony formation assay shown. (**C**) Quantification of triplicate colony formation assay staining intensity; same doses and concentrations as presented in (**B**). Data presented as mean ± SEM unless as otherwise indicated; *p < 0.05. Blots cropped for conciseness; full length images available in Supplementary Data.

To extend our results, we tested the effects of KTN3379, a human HER3 mAb that potently inhibits HER3 activation and downstream signaling^14^. Western blot analysis of cells treated with NRG demonstrated strong activation of the HER3-AKT axis. Treatment with KTN3379, however, was able to effectively abrogate HER3 and AKT activation in all cell lines, both in the absence and presence of NRG stimulation (Fig. 3A).

We next sought to extend these findings to a proliferation phenotype. As expected, stimulation with NRG led to significant proliferation in all three HNSCC cell lines tested; addition of KTN3379 was able to abrogate the NRG-driven proliferation (Fig. 3B,C). These data suggest that HER3 inhibition, both ligand-independent and ligand-dependent, is an effective strategy for HER3 mediated AKT suppression and tumor cell proliferation.

### Co-targeting HER3 and PIK3CA synergistically inhibited HNSCC cell growth *in vitro*

Given the previous findings, we hypothesized that by suppressing HER3 activity, KTN3379 would be able to suppress residual signaling through PI3K and would thus demonstrate synergistic anti-cancer activity when combined with BYL719.

**Figure 4:**
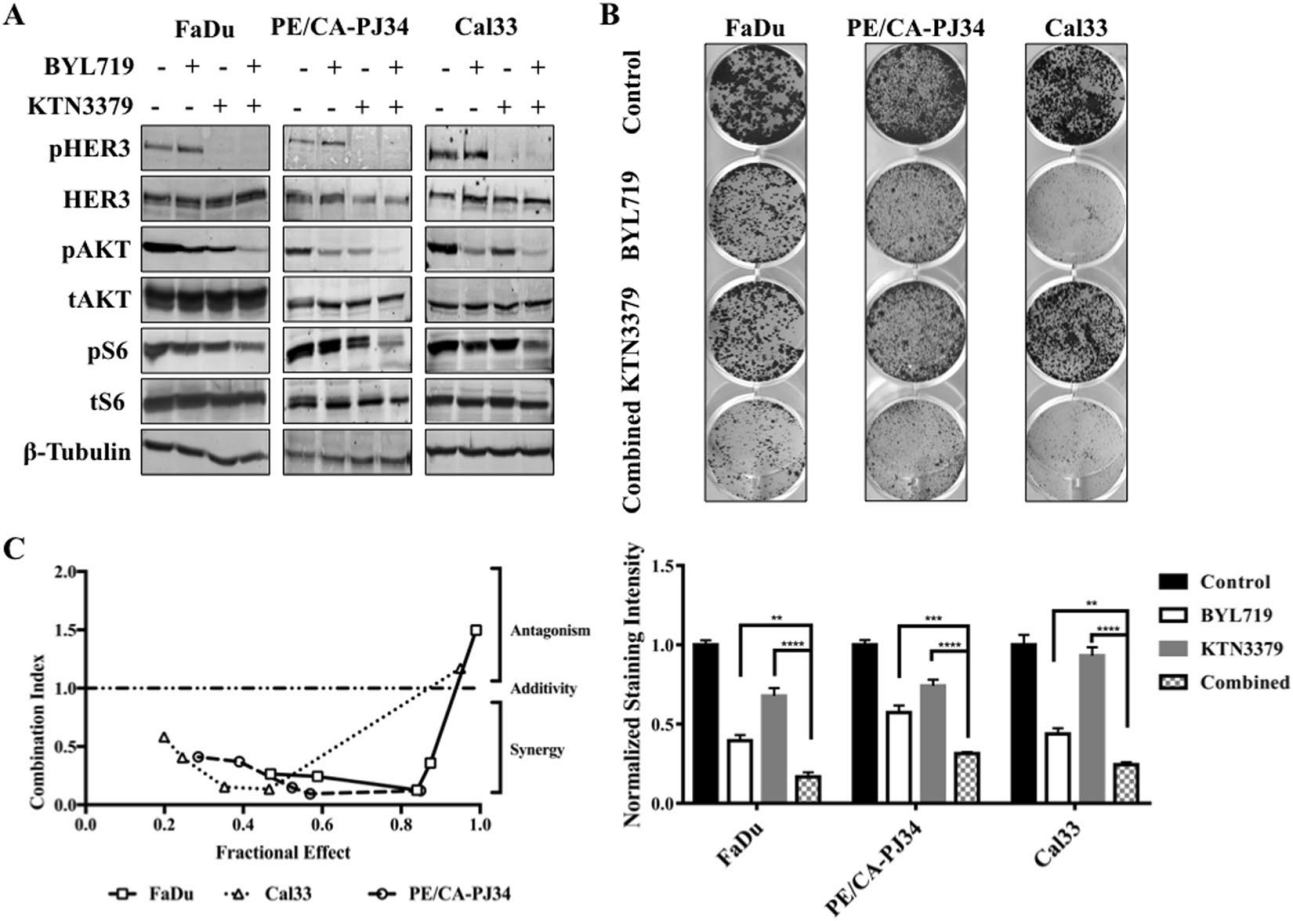
*PIK3CA*-HER3 dual targeting has synergistic effects in HNSCC. (**A**) Combination BYL719 (1 µM, 24 hours) and KTN3379 (100 nM, 1 hour) inhibits signaling along HER3-*PIK3CA*-AKT axis more effectively than single agent treatment with either compound in FaDu, PE/CA-PJ34, and Cal33 HNSCC cells. (**B**) BYL719 and KTN3379 demonstrate synergistic growth inhibition in colony formation assay. Significance assessed by one way ANOVA comparing BYL719 vs. Combined and KTN3379 vs. Combined presented in lower panel. (**C**) BYL719 and KTN3379 demonstrate synergy (Combination Index <1) in multiple HNSCC cell lines in growth assay. Drug added in fixed ratio (100:1). Data presented as mean ± SEM unless as otherwise indicated; *p < 0.05, **p < 0.01, ***p < 0.001, ****p < 0.0001. Blots cropped for conciseness; full length images available in Supplementary Data.

Addition of KTN3379 and BYL719 in all three HNSCC cell lines led to greater inhibition of HER3, AKT, and S6 than the inhibition observed with single-drug treatment with either BYL719 or KTN3379 (Fig. 4A). These molecular findings correlated with growth inhibition in colony formation assay - combination treatment with BYL719 + KTN3379 demonstrated enhanced growth inhibition compared to vehicle control and mono-therapy with either agent alone in all three tumor cell lines (Fig. 4B).

To determine if combination treatment with KTN3379 and BYL719 was additive or synergistic, we performed viability studies with the three cell lines exposed to combination treatment with KTN3379 and BYL719. Analysis of the viability results using the Chou-Talalay method revealed a combination index less than one for a majority of drug concentrations, indicating synergy (Fig. 4C & Sup. Fig. 4)^23^.

### Co-targeting HER3 and PI3K provides better tumor control than single-agent therapy in an *in vivo* model of HNSCC

**Figure 5:**
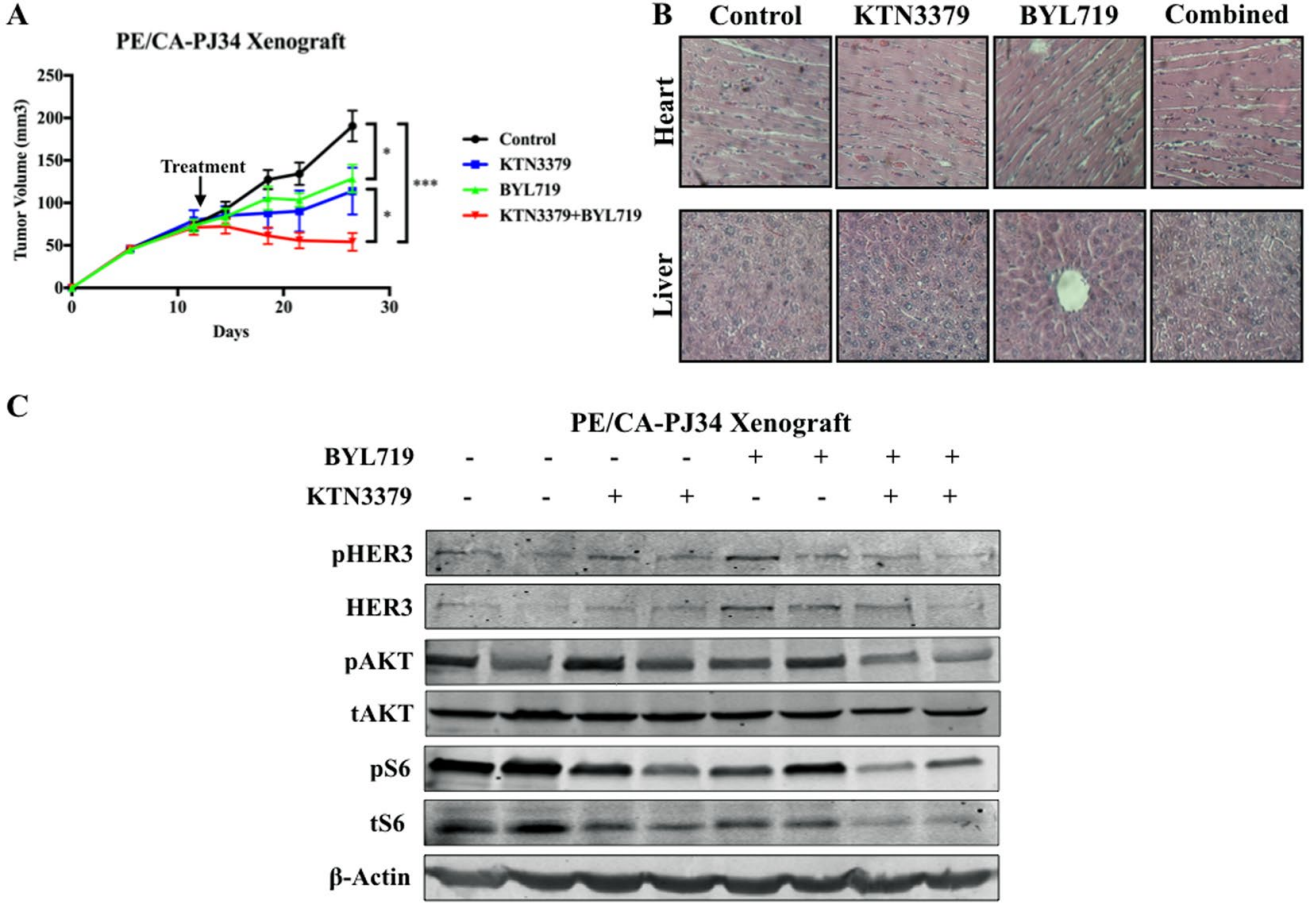
*PIK3CA*-HER3 dual targeting has synergistic effects *in vivo*. (**A**) Combination treatment with BYL719 (20 mg/kg, oral gavage) and KTN3379 (5 mg/kg I.P.) was better able to inhibit tumor growth relative to control and single-agent therapy in PE/CA-PJ34 xenograft model. n = 7 mice per treatment group. (**B**) Organs (heart and liver) from mice in each treatment group demonstrated grossly normal architecture. Representative images of lung, spleen, and kidney available in Sup. Fig. 4. (**C**) Combination treatment with BYL719 and KTN3379 inhibited HER3, AKT, and S6 expression and activation *in vivo*. Data presented as mean ± SEM unless as otherwise indicated; *p < 0.05; ***p < 0.001. Blots cropped for conciseness; full length images available in Supplementary Data.

In order to determine whether the effects of dual-inhibition observed *in vitro* would translate to *in vivo* models, PE/CA-PJ34 HNSCC cells were xenografted into mice which were randomly assigned to one of four treatment groups (control, KTN3379 only, BYL719 only, combination KTN3379 and BYL719) when tumors were palpable. Mice treated with the KTN3379-BYL719 combination had significantly smaller tumors than control and either mono-therapy treated groups (Fig. 5A). Observation of representative gross tissue specimens from all treatment groups (n = 7 per treatment group) demonstrated no cellular or tissue abnormalities in heart, liver, lung, spleen, or kidney (heart and liver - Fig. 5B; lung, spleen, kidney – Sup. Fig. 5).

Analysis of tumors treated with BYL719 in western blots revealed an increase in the expression and the activity (pHER3) of HER3. By comparison, tumor samples treated with combination KTN3379 and BYL719 had low pHER3/HER3 expression, as well as reduced signaling in downstream AKT and S6 (Fig. 5C). Densitometry analysis revealed non-significant trend-reductions in pHER3 and pAKT activity relative to control and single therapy groups, and a significant reduction in pS6 activity relative to control in the combination therapy group (Sup. Fig. 6).

## Discussion

Analysis of 151 HNSCC tumors by whole-exome sequencing at our institution demonstrated that 30.5% harbored at least one PI3K pathway gene mutation; 12.5% of all HNSCC specimens harbored a *PIK3CA* mutation^3^. The most common sites of *PIK3CA* mutations included H1047R/L (8 mutations total), E545K/G (4 mutations), and E542K (3 mutations), all of which represent previously reported hotspot mutation sites in other cancers such as prostate cancer, lung cancer, melanoma, and breast cancer^3,25–29^. PI3K pathway inhibitors are in early-phase clinical trials for solid tumors, including recurrent or metastatic HNSCC, highlighting the translational significance of this study.

HER3 is a very potent activator of the PI3K-AKT pathway. HER3 is known to have a direct phosphorylation binding site with the regulatory subunit, p85, through 6 consensus phosphotyrosine sites on its C-terminal tail^30,31^. Once phosphorylated, binding of HER3 with p85 activates the PI3K pathway, including activation of AKT^31,32^. HER3 expression has been shown to be upregulated in response to PI3K pathway inhibition in pre-clinical models of breast cancer^16,17,20^. PI3K has multiple subunits of which, BYL719 only suppresses the alpha subunit. We hypothesized that HER3 is able to drive residual signaling through the other subunits leading to persistent activation of proliferative signals. Thus, addition of KTN3379, would lead to more effective inhibition of the PI3K activator as a whole, leading to greater anti-cancer activity. Correspondingly, we found that combination treatment with BYL719 and KTN3379 was able to effectively suppress HNSCC growth *in vitro* and *in vivo* along with biomolecular activation of PI3K, AKT, and S6 in HNSCC^16–19^.

To begin addressing the hypothesis that the activity of other PI3K subunits were allowing for residual signaling, we treated HNSCC cells with LY290042, a pan-PI3K inhibitor, and KTN3379. We did not find additive suppression of AKT or S6 with combination treatment when compared with mono-agent therapy (Sup. Fig. 7). This provides early support for our hypothesis: the addition of a HER3 blocking agent would not be expected to add suppression if the downstream target, PI3K, were completely inhibited. More work is needed to test this hypothesis fully.

HER3 activity has been associated with resistance to other HER-family targeted inhibitors, erlotinib^33^. KTN3379, a human anti- HER3 mAb, was recently shown to significantly suppress tumor growth in HNSCC xenograft models and inhibit activations of HER3, EGFR, AKT and ERK *in vivo* when combined with cetuximab^34^. Several Phase 1b clinical studies investigating the antitumor activity, safety and pharmacokinetic profile of KTN3379 in advanced solid tumors in combination with targeted therapies are currently accruing patients. To date objective responses have been observed in patients with HNSCC and NSCLC cancers highlighting the potential for clinical application and translation of this work (NCT02014909; NCT02456701). Furthermore, an additional clinical study will evaluate tissue responses to KTN3379 in newly diagnosed HNSCC patients who are treated with KTN3379 prior to their surgical resection, and is ongoing at our institutions (NCT02473731). This study is expected to provide information on the effects of KTN3379 on biomarkers of activity including evaluating PI3K/PTEN status and phosphorylated-HER3, thereby informing future studies.

At present, we cannot determine which tumors will be sensitive to dual inhibition of HER3 and PIK3CA. All our experiments were conducted in HPV negative models; as such, we cannot predict the results of dual-targeting in the HPV positive environment. Additionally, though we used cell lines with representative *PIK3CA* statuses, we cannot preclude the possibility that other variables could further stratify sensitivity to dual-targeting into more precise subgroups. Recent data suggest that PTEN status may affect response to KTN3379^35^. Therefore, it is plausible that tumors harboring *PIK3CA* amplification, activating mutations, or PTEN loss may demonstrate differential response to HER3 inhibition. Further work is currently underway to explore this potential interaction^36–39^.

## Supplementary information

Supplemental Figures
