# Supplementary material for: HER3 targeting potentiates growth suppressive effects of the PI3K inhibitor BYL719 in pre-clinical models of head and neck squamous cell carcinoma
Source: Sci Rep. 2019 Jun 24;9:9130. doi: 10.1038/s41598-019-45589-y (PMC6591241; doi:10.1038/s41598-019-45589-y)
Supplement: Supplementary file 1 — Supplemental Figures [file 41598_2019_45589_MOESM1_ESM.pdf]

HER3 targeting potentiates growth suppressive effects of the PI3K inhibitor BYL719 in pre-clinical models of head and neck squamous cell carcinoma

Kara S. Meister[1*], Neal R. Godse[1*], Nayel I. Khan[1], Matthew L. Hedberg[1], Carolyn Kemp[1], Toni Brand[3], Sucheta Kulkarni[1], Diego Alvarado[2], Theresa LaVallee[2], Seungwon Kim[1], Jennifer R. Grandis[3], Umamaheswar Duvvuri[1]

[1]Department of Otolaryngology—Head & Neck Surgery, University of Pittsburgh Medical Center, Eye and Ear Institute, Suite 500, 200 Lothrop St. Pittsburgh, PA 15213

[2]Kolltan Pharmaceuticals, New Haven, CT

[3]Department of Otolaryngology—Head & Neck Surgery, University of California—San Francisco, San Francisco, CA

*The first 2 authors contributed equally to this work.

Running title: Targeting HER3 potentiates PI3K inhibition

Correspondence and/or reprint requests to:

Umamaheswar Duvvuri, M.D., Ph.D.
Department of Otolaryngology - Head and Neck Surgery
University of Pittsburgh Medical Center
Eye and Ear Institute
Suite 500
200 Lothrop St.
Pittsburgh, PA 15213
Phone: 412-647-2117
Fax: 412-647-2080
E-mail: duvvuriu@upmc.edu

Disclosure of Potential Conflicts of Interest:
Diego Alvarado and Theresa LaVallee were employees of Kolltan/CellDex Pharmaceuticals at the time of data collection.

This work does not represent the views of the US Government nor the Department of Veterans Affairs.

The following figures are (1) supplemental data and figures to support the above article and (2) a collection of full length blots for all electrophoretic data presented in the article.

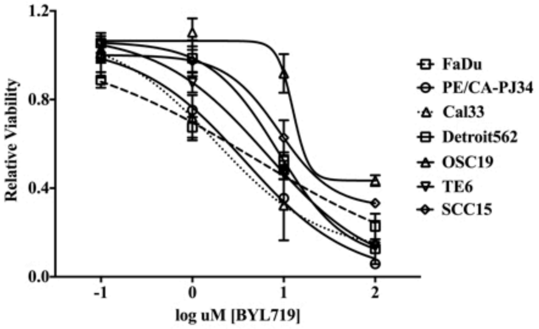

Cell Line	PIK3CA Status	PTEN Status	IC_{50} BYL719
**PE/CA-PJ34**	**Wild Type**	**Wild Type**	**4.15**
**FaDu**	**Amplified**	**Wild Type**	**4.22**
**Cal33**	**Mutant (H1047R)**	**Deleted**	**2.17**
**OSC19**	**Wild Type**	**Wild Type**	**23.01**
**SCC15**	**Amplified**	**Deleted**	**8.46**
**TE6**	**Amplified**	**Wild Type**	**5.81**
**Detroit562**	**Mutant (H1047R)**	**Wild Type**	**8.23**

Supplemental Figure 1. Summary of PIK3CA status, PTEN status, and BYL719 IC50 for 7 HNSCC cell lines.

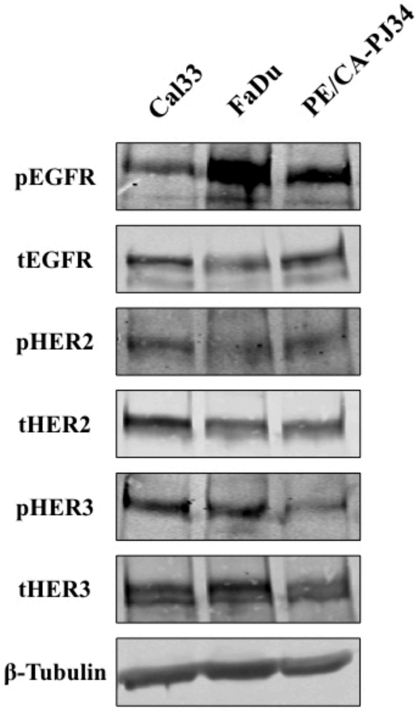

Supplemental Figure 2. Baseline expression and activation of HER-family receptors across three HNSCC cell lines.

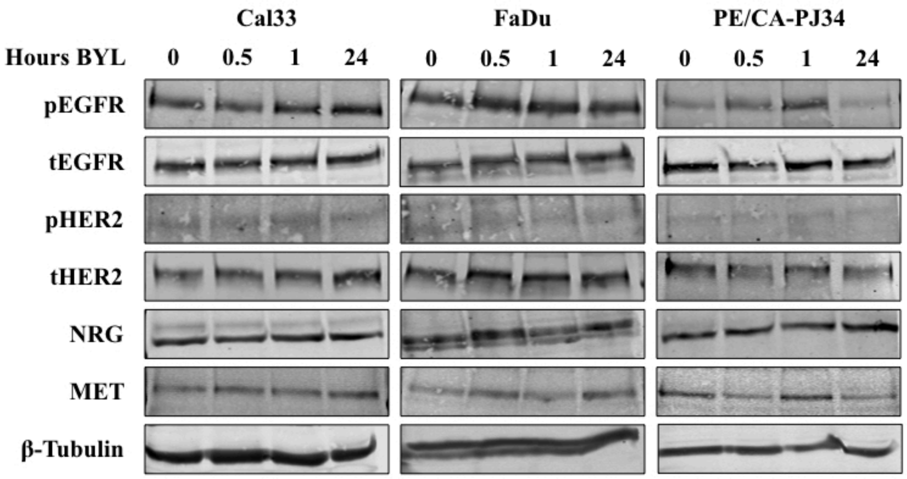

Supplemental Figure 3. HSNCC cell lines treated with BYL719 time did not demonstrate non-specific upregulation in other HER-family receptors, MET, or expression of NRG.

Combination Index: IC50 BYL719 + KTN3379		
**FaDu**	**Cal33**	**PE/CA-PJ 34**
**.29**	**.66**	**.36**

Supplemental Figure 4. Combination index (CI) values at the ED_{50} for three cell lines treated with combination BYL719 & KTN3379 in fixed, 100:1 ratio (CI < 1 indicates synergy).

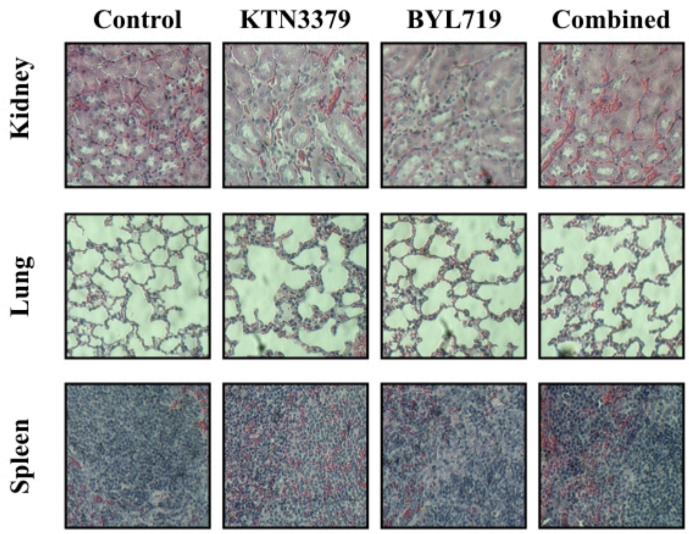

Supplemental Figure 5. Representative kidney, lung, and spleen tissue images from the organs of mice xenografted with PE/CA-PJ34 tumors treated with the indicated conditions.

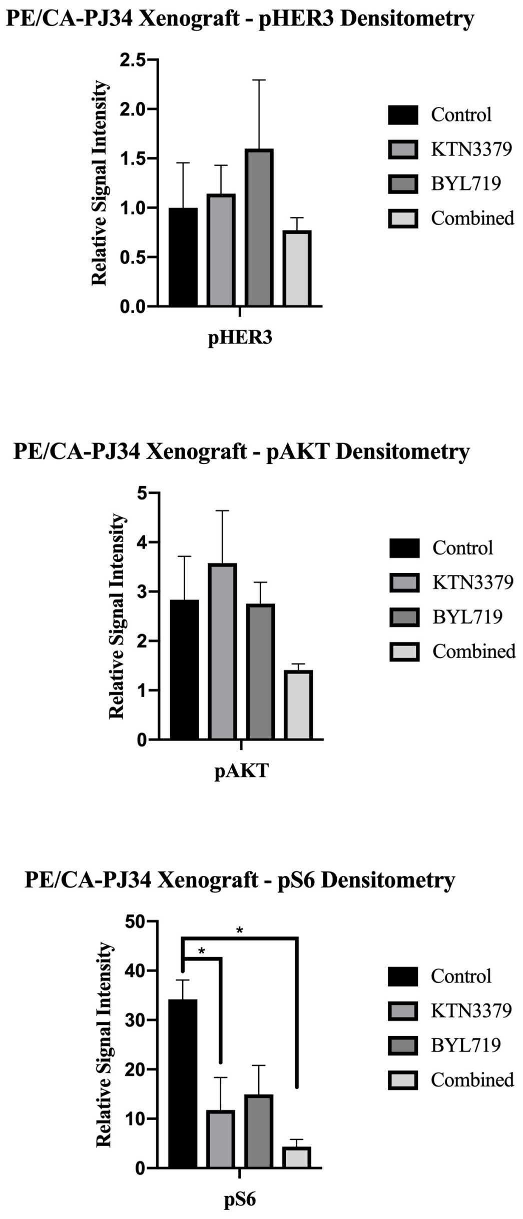

Supplemental Figure 6. Densitometric analysis of PE/CA-PJ34 Xenograft analysis. Relative signal intensity presented normalized to tubulin leels. *$p < 0.5$.

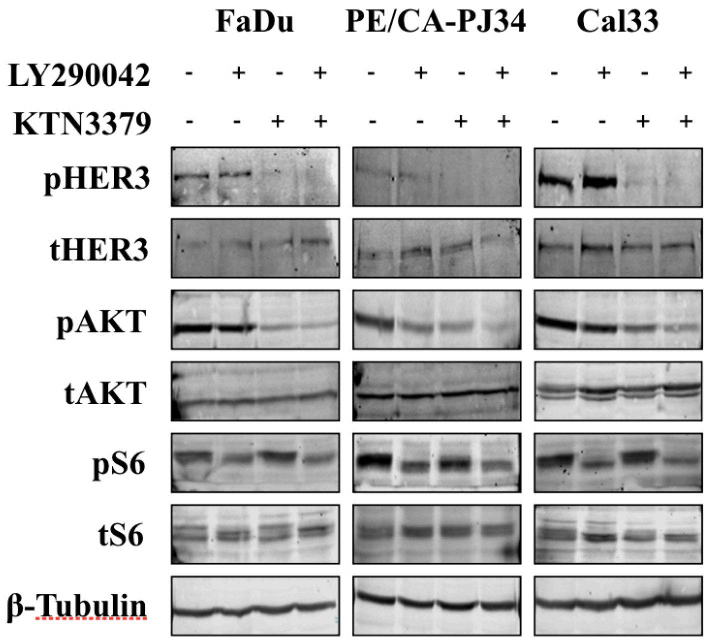

Supplemental Figure 7. Pan-PI3K inhibition with LY290042 did not produce additive or synergistic suppression of AKT or S6 with KTN3379 in HNSCC cell lines.

The remaining images are full length blots presented in the main article figures and supplemental figures of "HER3 targeting potentiates growth suppressive effects of the PI3K inhibitor BYL719 in pre-clinical models of head and neck squamous cell carcinoma" Each blot marked with the protein probed for, the molecular weight, and the figure in which the cropped image is presented.

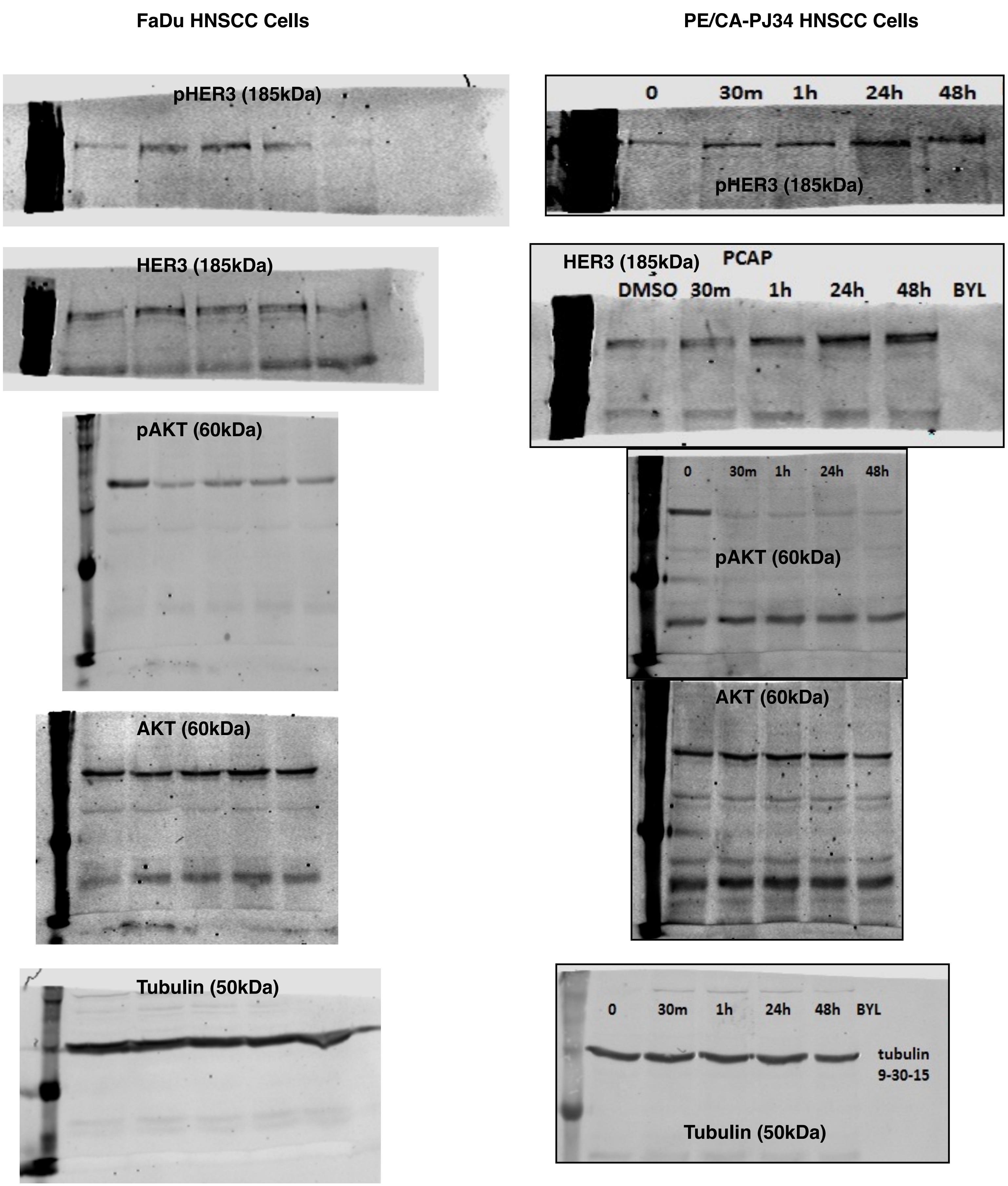

Full Length blots from Figure 1B. FaDu and PE/CA-PJ34 cells treated with time course of BYL as labeled Figure 1B

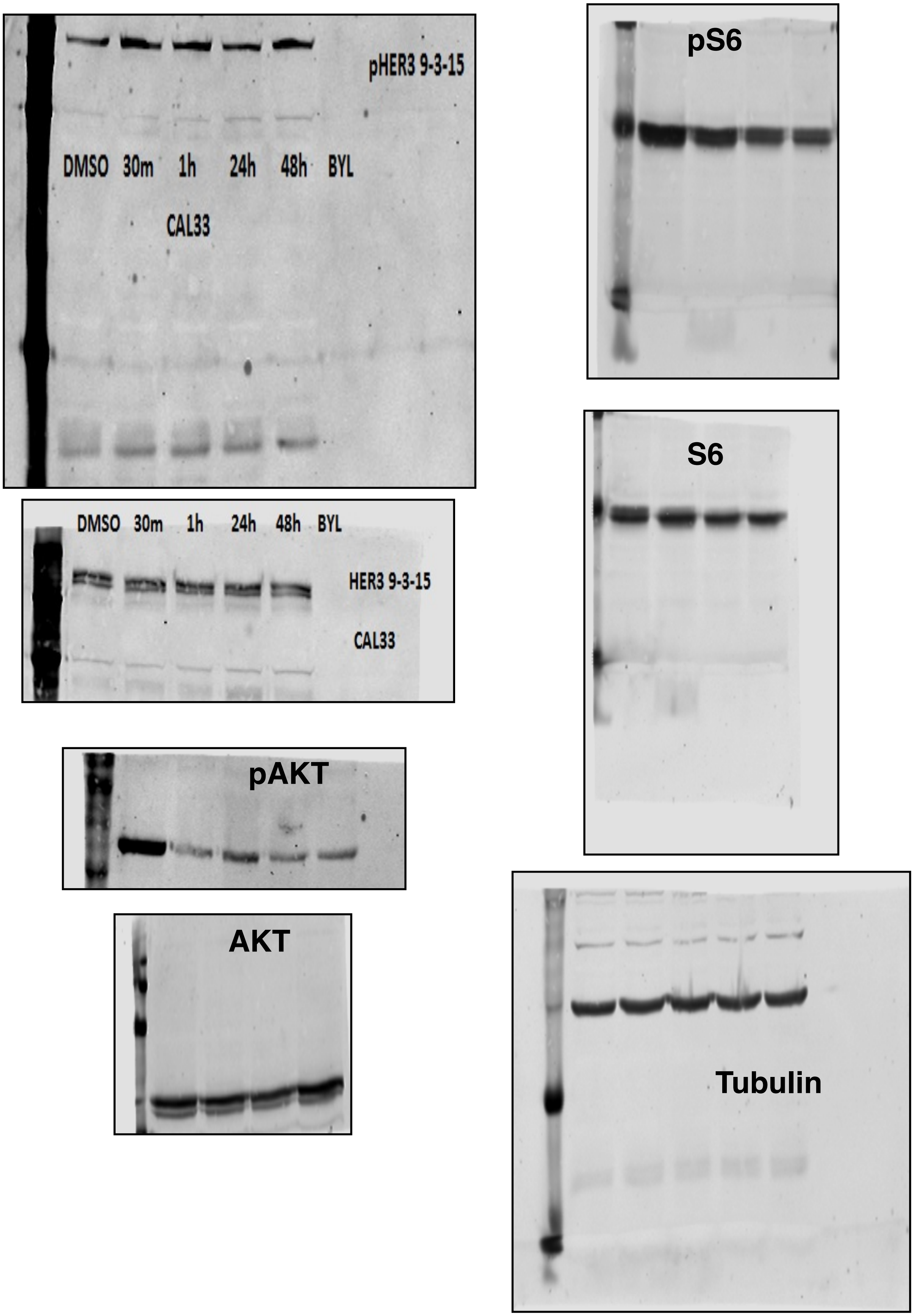

Uncropped gels from figure 1B - Cal33 cells with time course BYL719 treatment as labeled in figure 1B

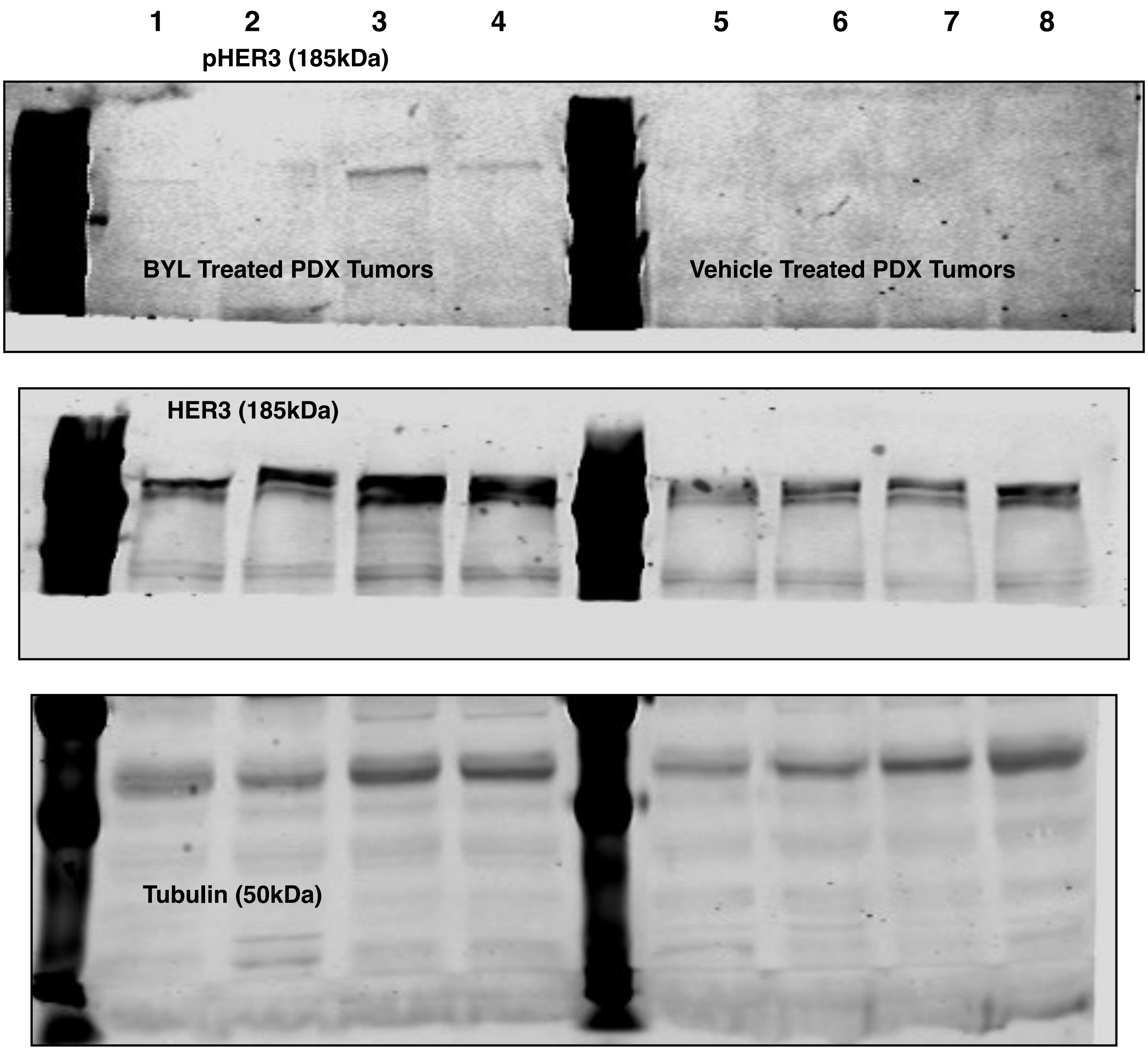

Full Length blots from Figure 1D. Vehicle treated PDX tumors (lanes 5, 6, 7, 8; lanes 7 and 8 presented in Figure 1D) and BYL treated PDX tumors (lanes 1, 2, 3, 4; lanes 3 and 4 presented in Figure 1D).

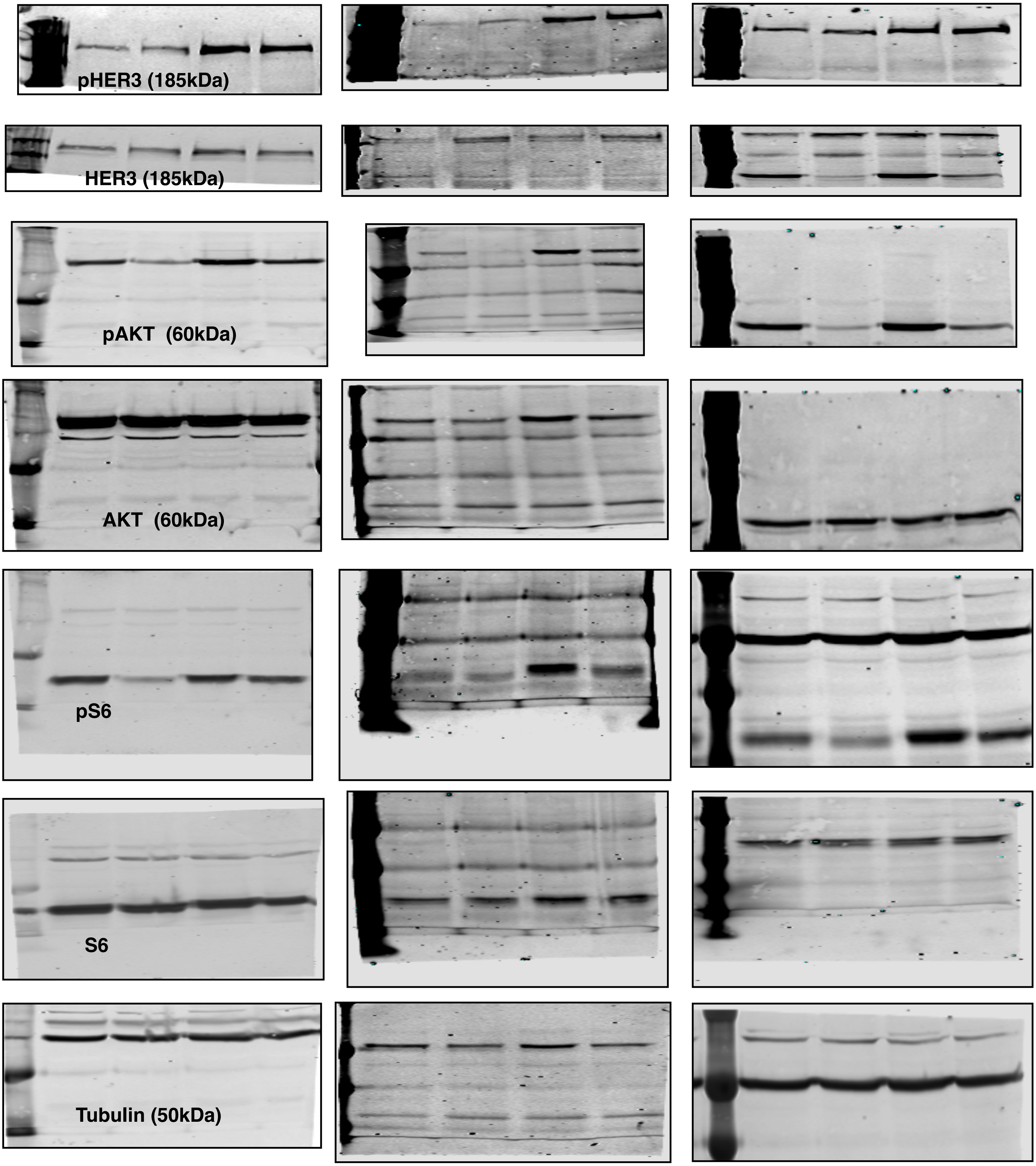

Full Length blots from Figure 2A. HNSCC cells treated with NRG and BYL (as indicated in Figure 2A). Columns from left to right: FaDu, PE/CAP-J34, Cal33.

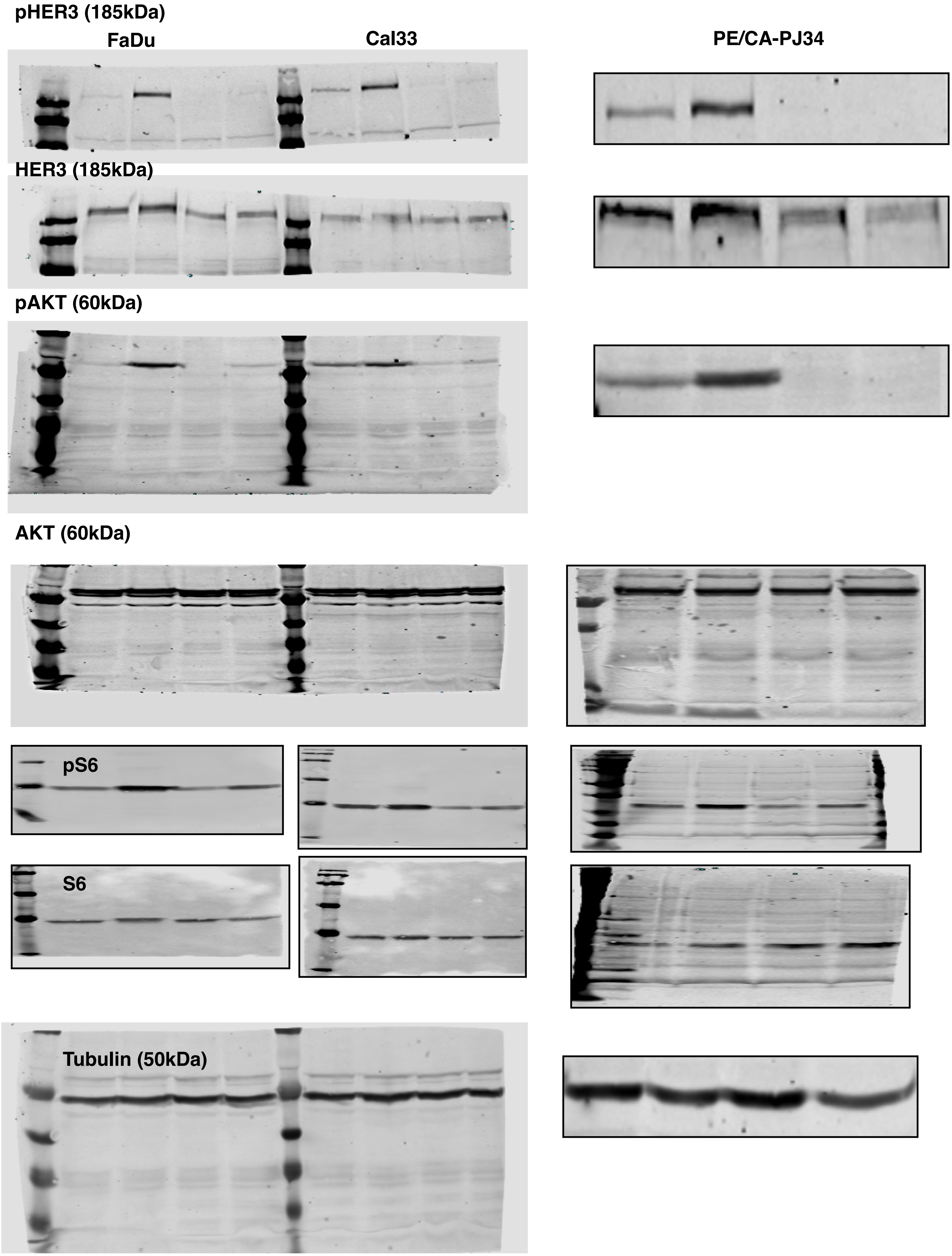

Full Length blots from Figure 3A. FaDu (Lanes 1-4), Cal33 (Lanes 5-8), PE/CA-PJ34 (right most column) treated with NRG and KTN3379 as indicated in Figure3A.

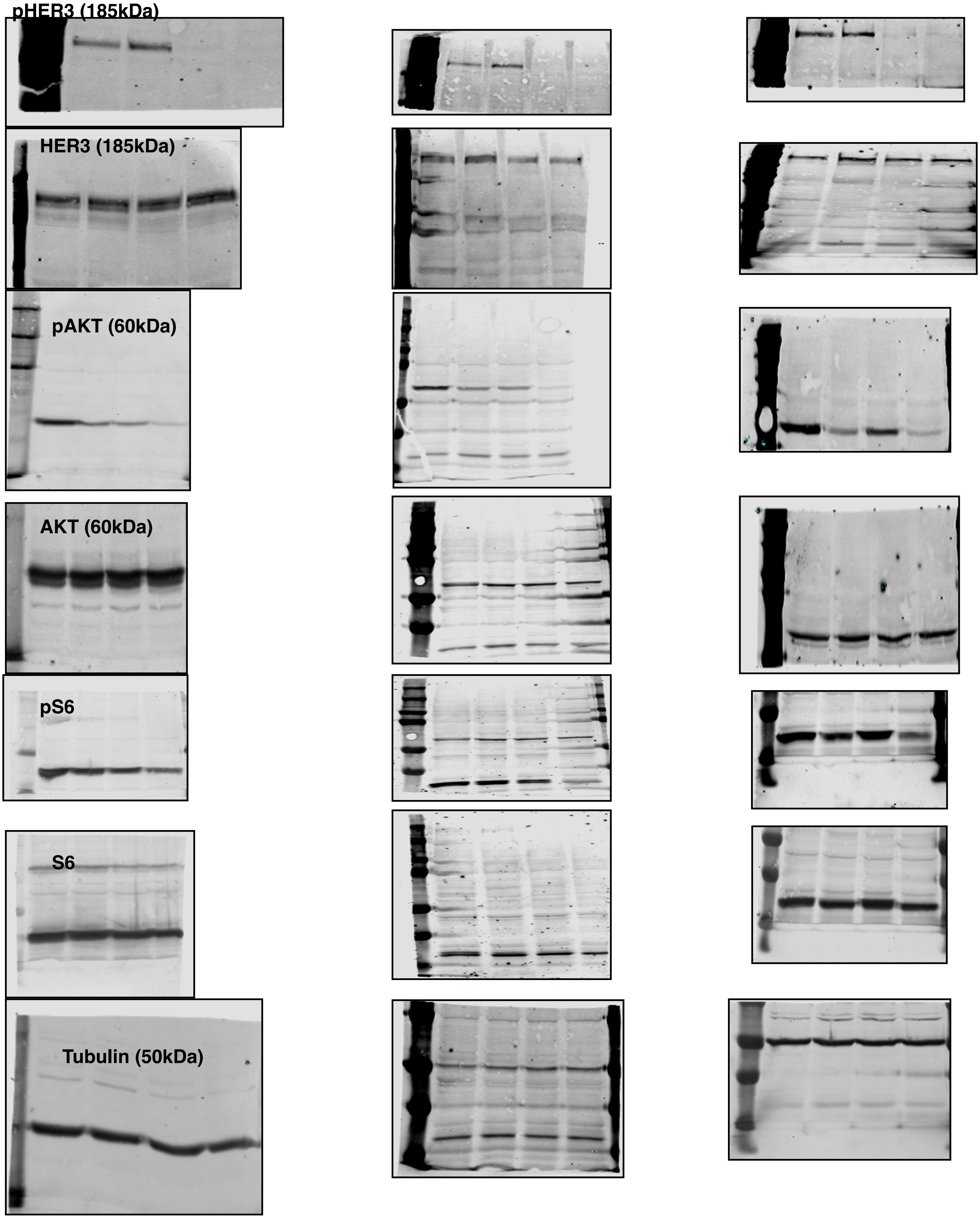

Full Length blots from Figure 4A. From left to right: FaDu, PE/CA-PJ34, and Cal33 HNSCC Cells treated with BYL719 or KTN3379 as indicated in Figure 4A.

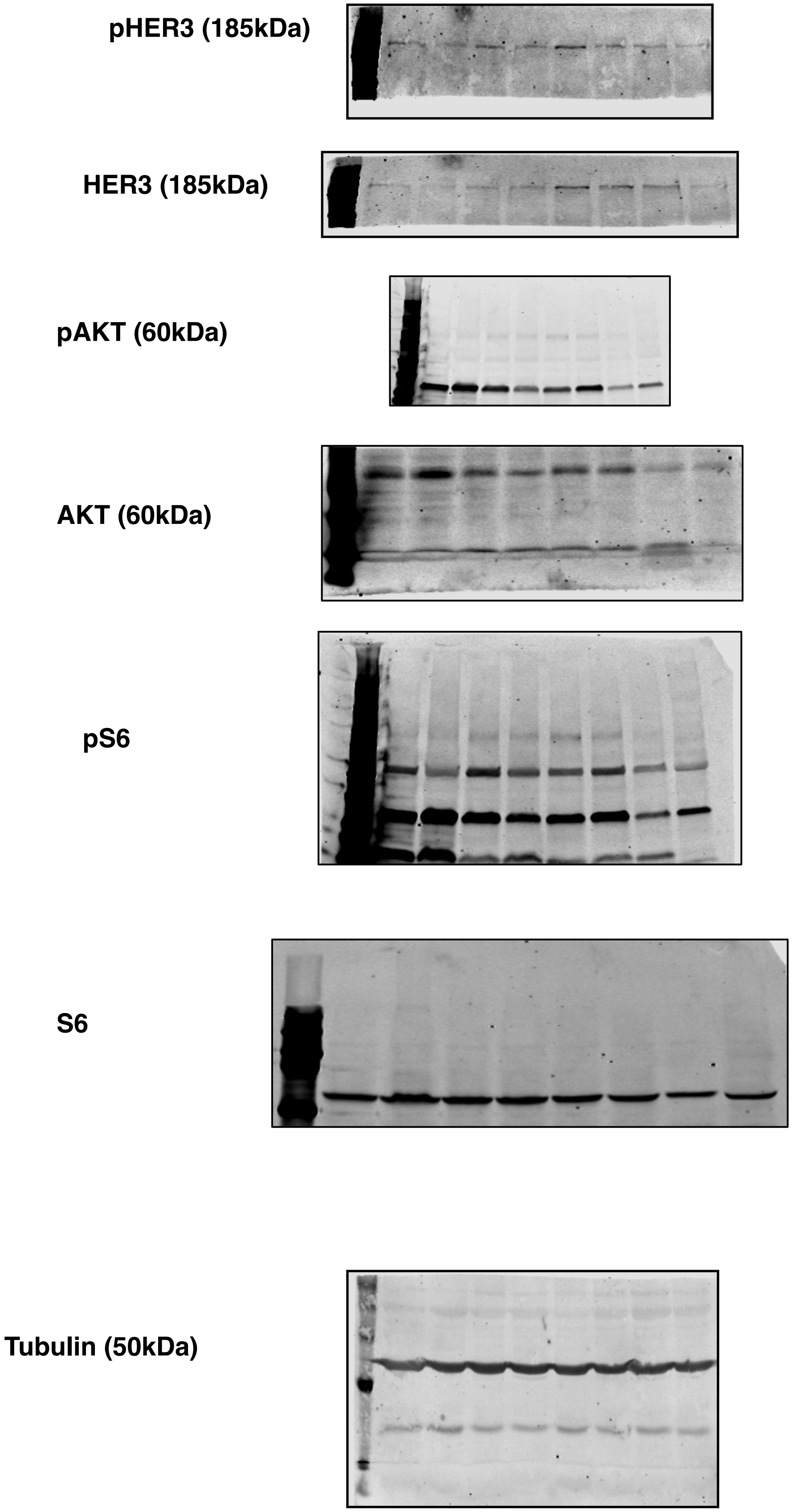

Full Length blots from Figure 5C. PE/CA-PJ34 HNSCC xenograft tumors treated with vehicle, KTN3379, BYL719, or combination.

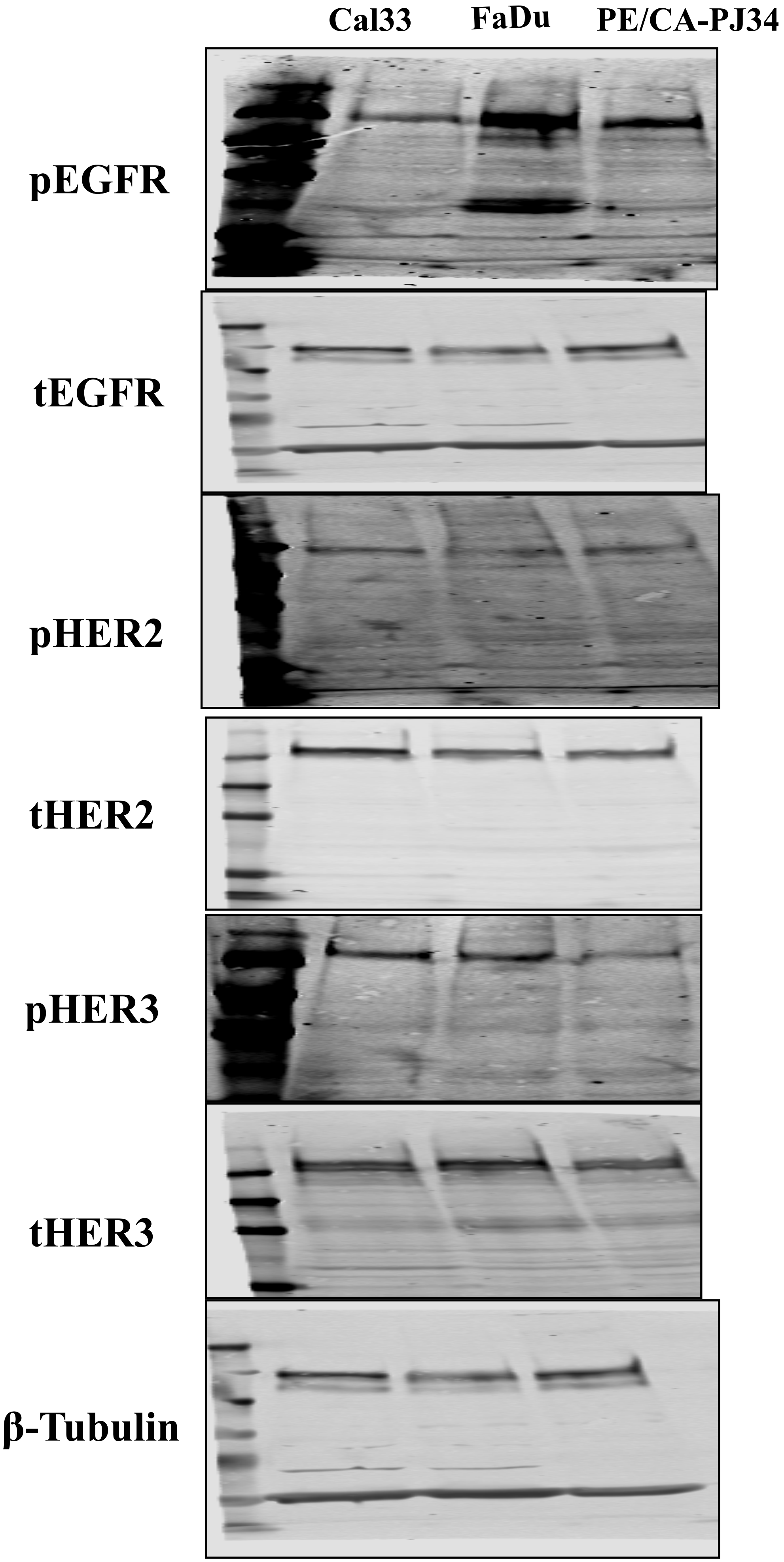

Full length blots from supplemental figure 2. Baseline expression of HER family receptors in, from left to right lanes 1-3, Cal33, FaDu, and PE/CA-PJ34 cells.

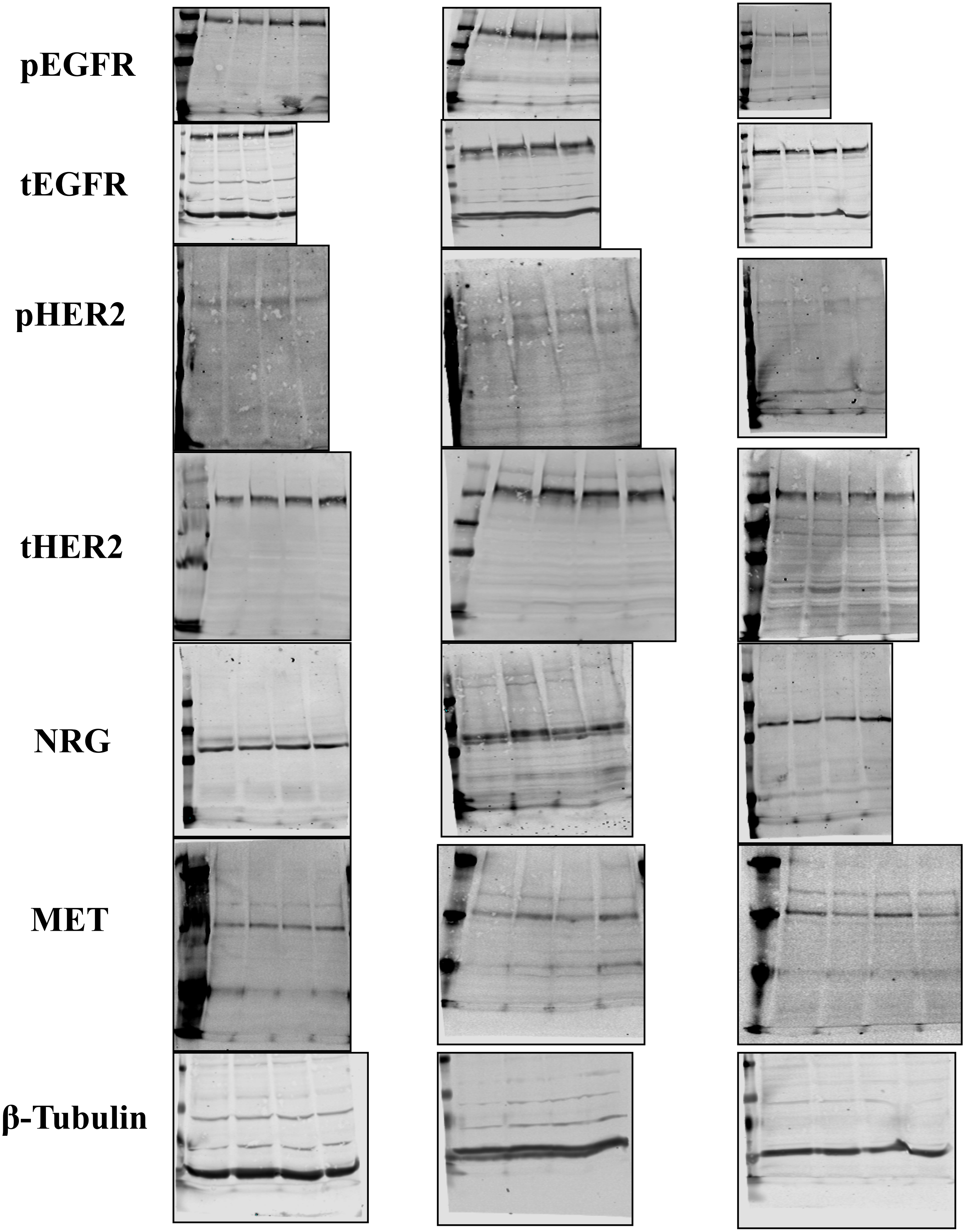

Full length blots from supplemental figure 3. From left to right: Cal33, FaDu, PE/CA-PJ34 cells treated with time course BYL719 as indicated in supplemental figure 3.

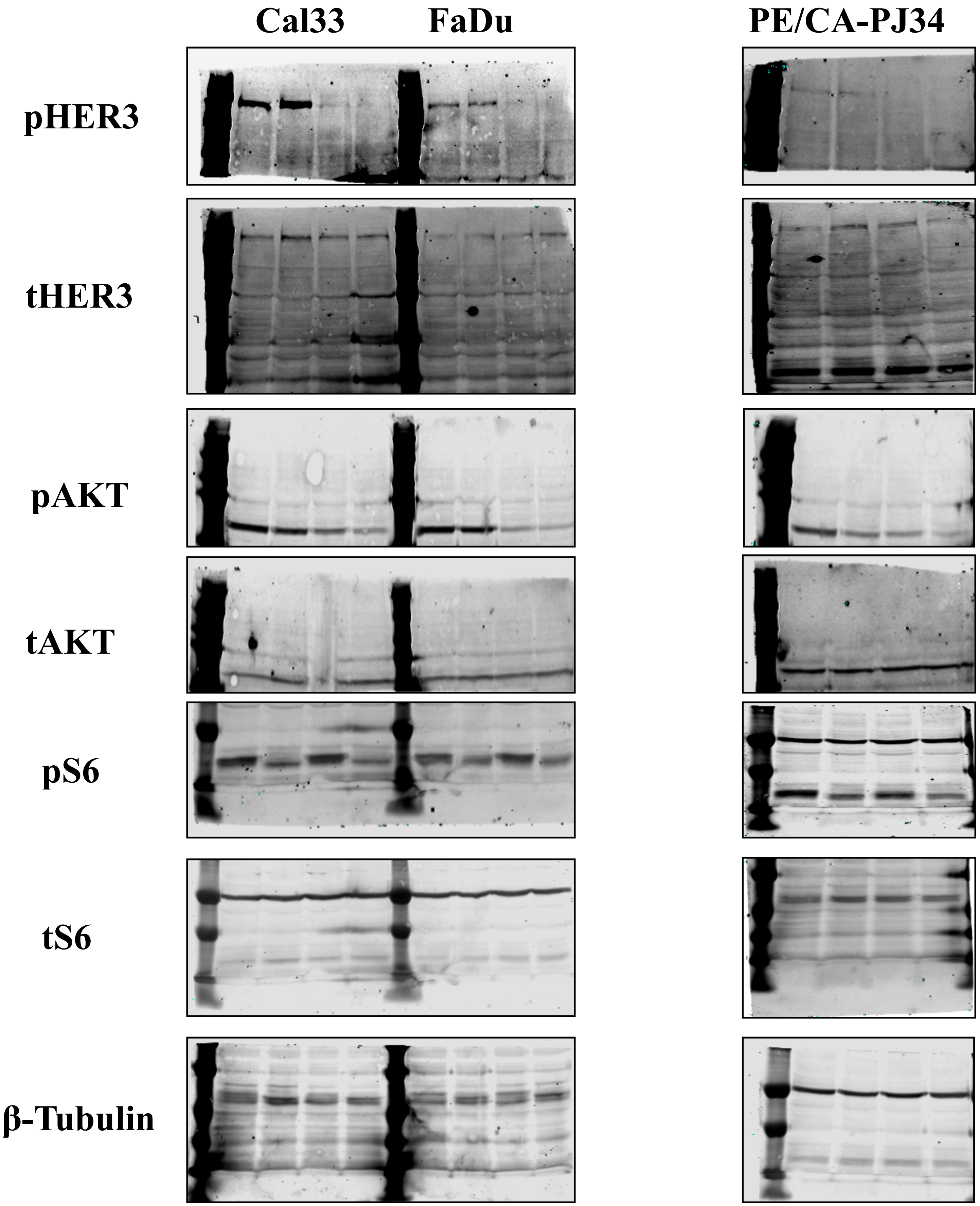

Full length blots from supplemental figure 6. From left to right: Cal33, FaDu, PE/CA-PJ34 cells treated with LY290042 and KTN3379 as indicated in supplemental figure 6.
